# The Munich Ankle Questionnaire (MAQ): a self-assessment tool for a comprehensive evaluation of ankle disorders

**DOI:** 10.1186/s40001-018-0344-7

**Published:** 2018-09-28

**Authors:** Frederik Greve, Karl Friedrich Braun, Veronika Vitzthum, Michael Zyskowski, Michael Müller, Chlodwig Kirchhoff, Peter Biberthaler, Marc Beirer

**Affiliations:** Klinik und Poliklinik für Unfallchirurgie, Klinikum rechts der Isar, Technische Universität München, Ismaninger Str. 22, 81675 Munich, Germany

**Keywords:** PROM instrument, Ankle PROM, Self-reported outcome measurement, Ankle, Validity: reliability, Responsiveness

## Abstract

**Background:**

There are many approved patient-related outcome measurement tools regarding ankle pathologies. However, there is none incorporating the range of motion (ROM) as an objective parameter. Most instruments focus on subjective parameters such as pain and impairment at work or daily living. Furthermore, the majority is only applicable to a specific pathology. Therefore, the objective of our study was to develop and validate the Munich Ankle Questionnaire (MAQ) as a universal self-assessment score including subjective and objective items.

**Methods:**

The established McGuire Score, Bray Score, Ankle Hindfoot Score (AOFAS) and Olerud and Molander Score were analyzed for relevant items and subscales. Items of interest were then condensed and allocated to the respective subscales of the MAQ. The final MAQ consists of 6 items addressing general and demographic data and 12 items addressing three domains: pain (3 items), work and daily living (5 items), movement and ROM (4 items). The evaluation of validity, reliability and responsiveness of the MAQ was performed in a prospective clinical study including traumatic as well as degenerative ankle pathologies.

**Results:**

In total, 148 patients (79 female, 69 male, median age 45 years) were included in the validation study. With intra-class correlation coefficients of at least 0.77, test–retest reliability was proven. Construct validity with a correlation coefficient of 0.82 and responsiveness with a correlation coefficient ranging from 0.42 to 0.47 were confirmed.

**Conclusion:**

The MAQ is a reliable and valid self-assessment measurement tool for the follow-up examination regarding subjective and objective parameters of traumatic and degenerative ankle pathologies. The MAQ has no limitation to specific disorders and allows a broad application.

**Electronic supplementary material:**

The online version of this article (10.1186/s40001-018-0344-7) contains supplementary material, which is available to authorized users.

## Background

Patient-reported outcome measurement (PROM) tools have been used increasingly as they are useful additive tools in daily clinical routine and patient physician interaction. The outcome measures can help to evaluate the individuals’ health status after a certain surgical procedure or to monitor the healing process after suffering from different pathologies [1, 2]. Furthermore resources can be saved as the patients themselves complete the PROM and no further investigator is needed. In addition to physician-based clinical examinations, PROMs serve to obtain further individual data regarding the long-term subjective satisfaction, as this aspect often does not correlate with the results of clinical evaluation [3]. According to a review of the literature, several commonly used questionnaires regarding ankle function could be identified [4–8]. In particular, the Ankle Hindfoot Score (AOFAS) [9], Olerud and Molander Score [4], Bray Score [6] and McGuire Score [10] are frequently used as PROMs for the lower extremity including the ankle. However, most of the validation studies focused only on a small spectrum of disorders. For instance, the McGuire Score was only used with patients who underwent ankle arthroplasty or ankle arthrodesis and the Olerud and Molander Score focuses on the postoperative function after treatment of multicomponent ankle fractures and has to be conducted by an investigator [4, 10]. The respective questionnaires should not be used for a wide spectrum of ankle pathologies. Martin et al. [8, 11] give an overview of existing ankle questionnaires and describe the presence of validity, reliability and responsiveness as mandatory criteria for proper test interpretation. Despite many available measurement tools, none of the scores include a scale to assess the range of motion (ROM), even though it serves as an important parameter to evaluate the postsurgical ankle function during rehabilitation and can be easily measured by the patient himself [12]. Until today, there is no single tool that combines all relevant factors and is furthermore valid, reliable and sensitive to clinical change [13, 14].

Therefore, this prospective study aimed to introduce and validate the Munich Ankle Questionnaire (MAQ) as a new universal measurement tool, which contains all relevant subjective and objective items and can be used as an instrument for follow-up examination in a patient collective with heterogeneous disorders without any limitations. For proper interpretation, we determined evidence for internal consistency, test–retest reliability, construct validity and responsiveness to include all required interpretation criteria in the validation process.

## Methods

### Development of the scoring system

All scales and items of the AOFAS, the Olerud and Molander Score, the Bray Score and the McGuire Score were analyzed for congruency in specific ankle measurements. To cover all relevant information, the most important and self-assessable items were finally condensed and allocated to an appropriate subscale.

The final Munich Ankle Questionnaire consists of 6 items addressing general and demographic information and twelve items addressing three domains: pain (3 items), work and daily living (5 items), movement and ROM (4 items). As a new aspect compared to preexisting ankle PROMs and for better understanding, the ROM measurement was illustrated as photographs. The highest achievable subjective value (pain, work and daily living) is 77 of 106 points. The maximum value of the objective domains (movement and ROM) is 29 out of 106 points. This indicates a subjective–objective ratio of almost 2:1. The overall score is then converted into a percentage scale for better interpretation. The best possible result is 100% and a value of 0% represents the poorest possible result. Table 1 gives a recommendation for outcome grading in dependence of the overall MAQ score. Additional file 1 demonstrates the complete MAQ.

**Table 1:** Outcome grading of in dependence of the MAQ result

MAQ result (%)	Outcome grading
90–100	Excellent
70–89	Good
50–69	Moderate
< 50	Poor

### Patient collective

Between March 2016 and June 2017, a randomly selected cohort of 162 patients from our outpatient clinic who presented heterogeneous disabilities of the ankle including acute fractures as well as degenerative diseases were asked to participate the validation study. Before enrollment of the patients, written informed consent was obtained. In case of dementia, psychiatric diseases or other cognitive diseases patients were excluded.

The study protocol was approved by the local ethics committee (voting 5631/12) and carried out in accordance with the World Medical Declaration of Helsinki.

### Testing and evaluation of measurement qualities

#### Floor and ceiling effects

The presence of floor and ceiling effects impacts score interpretation. If a questionnaire is not able to detect poor or high results in patients with obvious clinical signs for a worse or superior condition, floor and ceiling effects may be present resulting from an incomprehensive scale.

Floor and ceiling effects are defined if more than 15% of the patients achieve the highest or poorest possible test result [15, 16].

#### Internal consistency

Internal consistency describes the degree of correlation between different items on the same test, thus measuring the same construct [17].

#### Test–retest reliability

Test–retest reliability describes the capability of score to measure the same test result within a period where the health status or the individual’s condition can be expected to be stable [11]. Thus, test–retest reliability evaluates the stability of a score. The patients have to complete the same questionnaire at least one more time. However, the period between the repeated measurements should not be too short to prevent recall of the tested items. In this study, the patients were asked to complete the second MAQ 2 weeks after initial testing. The tests were handed out during the first visit in our outpatient clinic along with a stamped addressed envelope for the return or the questionnaire.

#### Construct validity

Construct validity assesses how the scores on the respective instrument relate to other preexisting PROMs and in what degree they are consistent based on the assumption that the PROM validly measures the construct to be measured [11, 18]. Construct validity was assessed by correlating the subscales of the MAQ with the matching subscales of preexisting measurement tools, which ideally had evidence for construct validity, test–retest reliability and responsiveness. The subscale pain was correlated with the Foot and Ankle Outcome Score (FAOS) [5, 19], the subscale work and daily living was correlated with the Foot and Ankle Ability Measure (FAAM) [8]. All the correlating scores can be used for follow-up examinations of ankle pathologies and have at least some evidence for score interpretation [11, 14]. The subscale ROM which was completed from the patients themselves in a first step was then correlated with the results of a clinical examination assessing the exact degree of possible dorsiflexion and plantar flexion. The patients were asked to complete the respective questionnaire during their first visit in our outpatient clinic.

#### Responsiveness

A PRO instrument is responsive if it detects changes in an individual’s status over time [11, 18]. To determine responsiveness a global perceived effect (GPE) score was used. This consists of one question on the patients’ subjective status regarding improvement or worsening of their ankle function during the past 4 months. The list of potential answers contained seven categories (much better [+ 3], better [+ 2|, somewhat better [+ 1], no change [0|, somewhat worse [− 1], worse [− 2], and much worse [− 3]) for each subscale of the MAQ. Four months after initial testing, the MAQ was sent by mail with a GPE score to the patients. A period of 4 months is long enough for a potential change in the patients’ condition and not too short for potential recall of the single items. In case of missing scores, patients were reminded by phone call.

**Fig. 1:**
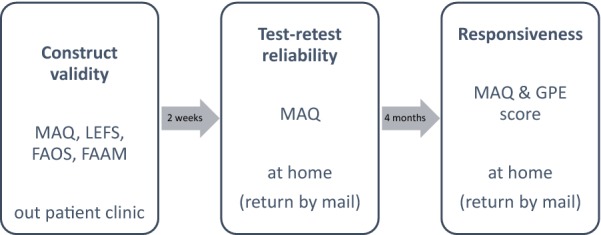
Illustration of the workflow for assessment of construct validity, test–retest reliability and responsiveness with the respective questionnaires at several time points including the setting of completion

Figure 1 depicts the workflow and the respective questionnaires which had to be completed at several time points.

#### Correlation of the MAQ with preexisting ankle scores

To evaluate the correlation with preexisting PROMs, the result of the MAQ as a new measurement tool was correlated with the results of preexisting tools. For correlation, we chose the FAOS [5], FAAM [8], and Lower Extremity Functional Scale (LEFS) [7] as frequently used PROMs with evidence for validity, reliability and responsiveness.

### Statistics

Statistical analysis was performed using the Software Sigmastat, Version 3.5, Systat Software GmbH, Erkrath, Germany.

In this study, we calculated the Cronbach’s *α* for all subscales to proof internal consistency for the respective items. A value above 0.7 indicates internal consistency [15, 20]. Intraclass correlation coefficients (ICCs) were calculated to determine test–retest reliability. Values greater than 0.7 assume positive reliability [11, 15, 20, 21]. For determination of construct validity, Pearson correlation coefficients (PCCs) were calculated and positive construct validity was assumed with a PCC above 0.7 [21–23]. Spearman correlation coefficients (SCCs) were calculated to identify the presence of responsiveness. According to previous studies, an SCC between the change in the MAQ score and the GPE score of at least 0.4 was assumed to indicate high responsiveness [21, 23, 24]. To determine the correlation of the MAQ with preexisting ankle scores, PCCs were calculated and correlation was depicted by a linear regressions analysis. PCCs higher than 0.7 indicate positive correlation.

## Results

### Patient collective and study design

**Fig. 2:**
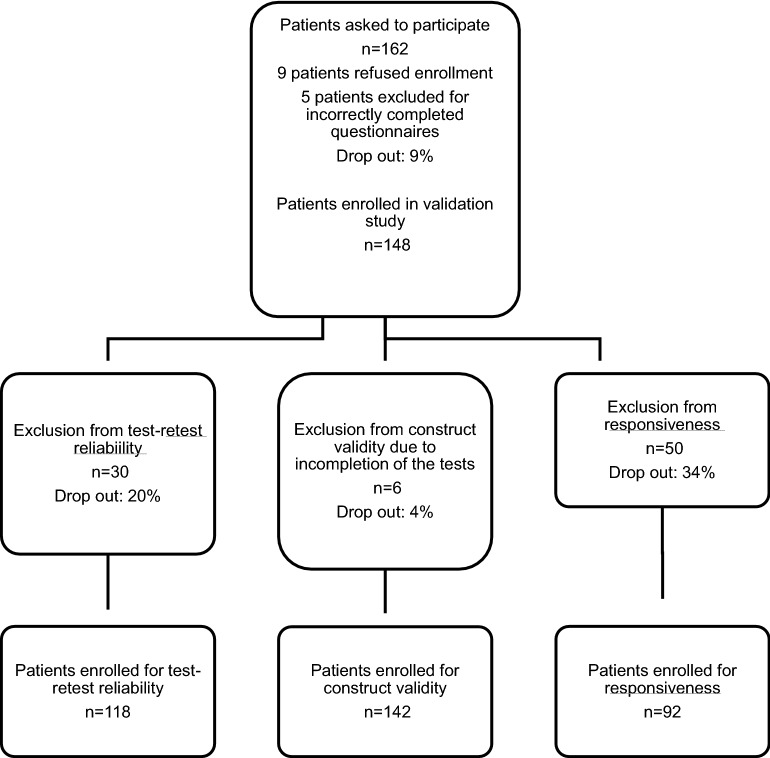
Illustration of the patient collective with drop-out rates for construct validity, test–retest reliability and responsiveness. Patients were excluded due to incompletion or no postal return of the questionnaires

Between March 2016 and June 2017, among in total 162 patients, 148 patients could be included in this prospective study for evaluation of validity, reliability and responsiveness of the MAQ after written consent was obtained. At initial consultation in our outpatient clinic, the patients were asked to complete the MAQ, LEFS, FAOS and FAAM. Furthermore, the physician performed an objective measurement of the ROM of both ankles using a goniometer. 148 individuals (53% female, 47% male, median age of 45 years, SD of 16.48) could be included and completed the majority of the items to ensure interpretation of at least single subscales. Majorly uncompleted (missing items in all subscales) and obviously misinterpreted questionnaires were excluded. Table 2 highlights the wide spectrum of the individuals’ ankle pathologies. Patients suffered from acute injuries like fractures as well as from rather chronic diseases such as osteoarthritis and cartilage disorders. All individuals suffered from isolated ankle pathologies. For assessing reliability, the patients were asked to complete another MAQ 2 weeks after the initial consultation. We received 118 completed questionnaires after an average period of 18 days. Thirty patients did not return the questionnaires and had to be excluded for assessment of reliability. For measurement of responsiveness, another MAQ with an additional GPE score was sent to the patients for completion almost 4 months after initial consultation (mean 5 months). Ninety-two patients completed the questionnaires correctly and 50 individuals had to be excluded due to refusal to participate in further steps or not responding. Figure 2 depicts the study profile with the respective dropouts.

**Table 2:** Overview of all ankle disorders of the enrolled patients

Diagnosis	Total *n* = 148	Women *n* = 81	Men *n* = 67
Malleolar fracture (Denis–Weber B)	32	16	16
Malleolar fracture (Denis–Weber C)	5	4	1
Maisonneuve fracture	3	1	2
Tibial pilon fractures	6	2	4
Fractures of the talus	7	3	4
Bimalleolar fracture	10	8	2
Trimalleolar fracture	22	18	5
Fracture of the medial malleolus	3	0	3
Osteoarthritis	9	4	5
Non-union of the medial malleolus	1	0	1
Ankle distorsion/sprain	20	7	13
Osteochondrosis dissecans of the talus	9	7	2
Tear of the lateral ligament	8	6	2
Tear of the tibiofibular syndesmosis	7	2	5
Chronic instability	1	0	1
Scarification of muscle tissue	1	1	0
Impairment of wound healing	1	1	0
Unspecific pain	3	1	2
Total	148	81	67

### Floor and ceiling effects

Five individuals (3% of the patient collective) achieved the highest possible score of 100% and none of the individuals obtained the poorest result. Due to the fact that less than 15% achieved the highest or the worst possible result, there is no evidence for floor or ceiling effects.

### Internal consistency

Each subscale of the MAQ was analyzed for internal consistency by calculation of the Cronbach’s *α*. The lowest calculated value of all subscales was 0.87 and the highest value was 0.91 (Table 3). This indicates a high internal consistency for all items in the respective subscales.

**Table 3:** Illustration of the mean test results and the mean retest results for each subscale of the MAQ

	Test mean (SD)	Retest mean (SD)	ICC	Cronbach’s *α*
MAQ total	75.91 (19.47)	78.92 (19.49)	0.75	
Pain	21.4 (6.2)	21.83 (6.13)	0.8	0.89
Work and daily living	32.75 (11.18)	34.59 (10.12)	0.79	0.91
Movement and ROM	19.81 (6.48)	21.09 (6.08)	0.77	0.87

For determination of test–retest reliability, ICCs of the subscales were calculated. For assessment of internal consistency, Cronbach’s *α* was calculated. Values of Cronbach’s *α* higher than 0.7 indicate evidence for internal consistency

### Test–retest reliability

Two weeks after the first visit, another MAQ was completed and correlated with the initial questionnaire. The highest ICC was 0.8 and the lowest calculated ICC was 0.75 (Table 3). With all coefficients being higher than 0.7, test–retest reliability is proven.

### Construct validity

**Fig. 3:**
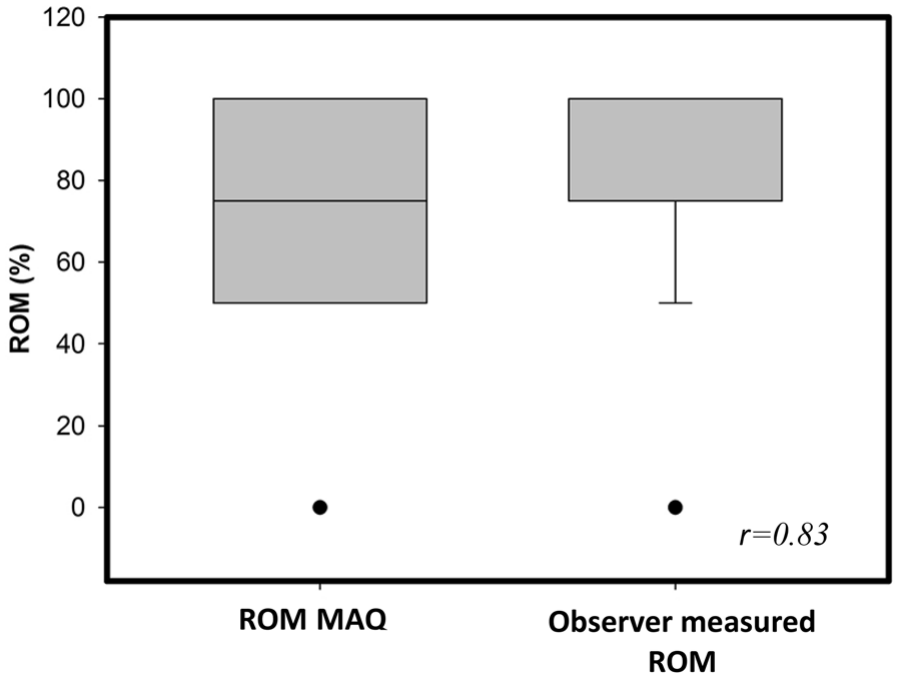
The graph presents the validation results of the ROM in a percentage scale. The first box shows the result of the MAQ. The second box depicts the objectively observer measured ROM. The calculated Pearson coefficient was *r *= 0.83

Assessment of construct validity was performed by correlating the subscales of the MAQ with the matching subscales of the FAOS (pain, work and daily living and movement) and the FAAM score (work and daily living and movement). The ROM subscale was furthermore correlated with the objectively measured degree of dorsiflexion and plantar flexion (Fig. 3). A PCC of at least 0.82 was calculated for all subscales (Table 4).

**Table 4:** The table contains the calculated PCCs for evaluation of construct validity

MAQ		FAOS			FAAM		Measured	
		Pain	Activities of daily living	Functional sports and recreational activities	Activities of daily living	Sports	ROM	ROM and MAQ movement
Pain		0.83	–	–	–	–	–	–
Work and daily living		–	0.85	–	0.85	–	–	–
ROM		–	–	–	–	0.82	0.83	–
Movement and ROM		–	–	0.82	–	–	–	0.85

The subscale “pain” was correlated with the matching subscale of the FAOS. “Work and daily living” was compared with the subscale “activities of daily living” of the FAOS and FAAM. The objectively measured degree of dorsal and plantar flexion was correlated with the results from the “ROM” subscale that were achieved by use of the illustrated dorsal flexion and plantar flexion. Furthermore, the PCC between “ROM” and the subscale sports from the FAAM was calculated. Finally, the overall result of “movement and ROM” was correlated with the subscale “functional sports and recreational activities” of the FAAM and the objectively measured ROM. PCCs higher than *r* = 0.7 indicate evidence for construct validity for all subscales of the MAQ

### Responsiveness

For evaluation of responsiveness, a MAQ with an additional GPE score for detection of status improvement or worsening regarding each subscale was sent to the patients 4 months after initial consultation. The SCC was 0.42 for pain, 0.47 for work and daily living and 0.42 for movement and ROM.

### Correlation of scores

**Fig. 4:**
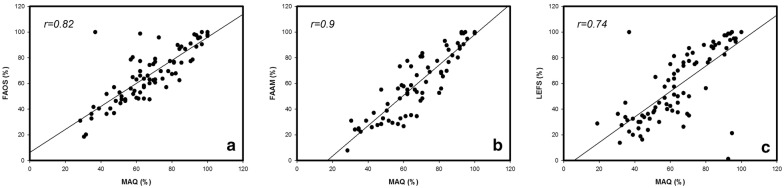
The graphs present the regression analysis of the MAQ with the FAOS (**a**), FAAM score (**b**) and LEFS (**c**). Pearson coefficients were *r* = 0.82 (MAQ vs. FAOS), *r *= 0.9 (MAQ vs. FAAM) and *r *= 0.74 (MAQ vs. LEFS). High PCCs indicate a correlation between the MAQ and the preexisting scores and identify the MAQ as a comprehensive ankle questionnaire. Furthermore, a representation of the existing scores in the new designed MAQ as a universal measurement tool can be assumed

The comparison of the achieved points of the FAOS, LEFS and FAAM and the calculated points from the MAQ is presented in Fig. 4. A PCC of at least 0.74 and 0.9 as maximum indicates for a high representation of all single scores in the new constructed MAQ as one single universal questionnaire.

## Discussion

It is reported that the increasing use of PROMs can lead to better integration of the patients into the healing process and to enhance shared decision-making [25]. For example, Ayers et al. mention the use of PROMs as tools to monitor the individual symptoms of patients suffering from chronic knee pain. The authors investigated retrospectively a cohort of patients who underwent total knee arthroplasty (TKA). All individuals completed a PROM before operation, as this is a standard procedure in many US clinics. The majority of the patients obtained a poor test result indicating a high subjective burden. Analyzing the data of PROMs may, thus, be helpful to critically evaluate if operative procedures such as TKA are necessary and when the potential of conservative treatment might be exhausted [26, 27]. By wide spread use, the introduced MAQ could serve for the same purpose regarding patients suffering from ankle disorders because it allows the assessment of the subjective (pain) and objective (movement and ROM) patient status.

Furthermore, PROMs can be used to detect potential side effects during a long-term therapy or to identify pathology connected high burdens, which are reported by patients after completing a PROM [28–30]. For example, Basch et al. [28] used an online PRO instrument to detect severe side effects of patients undergoing chemotherapy. The patients completed the questionnaires at home or during clinic visits in the waiting area. When a patient reported a severe toxicity, the treating physician was automatically informed by email. Oncologic treatment was then immediately adjusted. During daily routine with limited time for each patient, these important issues could be overseen easily.

In addition, PROMs can facilitate the access to research data in the field of orthopedics because the highest level of evidence cannot be achieved by randomized controlled trials (RCTs) concerning the comparison of non-operative and operative treatment procedures. As an alternative to RCTs, PROMs can be used to generate fracture registers including patients with conservative and operative treatment. Even immobile patients can be included.

Compared to other studies, the validation of the MAQ was performed with patients suffering from various ankle pathologies (Table 2). For instance, the evidence for score interpretation of the frequently used FAOS score and Foot and Ankle Disability Index (FADI) was collected in a patient collective that underwent lateral ankle ligament reconstruction [5] or suffered from chronic ankle instability [31]. Strictly speaking, these instruments are limited to ligamentous pathologies. By involving different diagnoses, the MAQ is a questionnaire appropriate for a wide range of ankle disorders. This distinguishes the MAQ from already existing ankle questionnaires.

The information acquired from a self-reported outcome instrument is only useful if evidence for internal consistency, validity, reliability and responsiveness was obtained in the respective validation study [13]. A meta-analysis by Button et al. [14] identified only 8 out of 49 foot and ankle scores with a performed validation study. For a proper interpretation of the MAQ, we required the determination of all necessary criteria.

The calculation of Cronbach’s *α* indicated internal consistency with a high extent of homogenous items in the respective subscale measuring the same construct (Table 3) [15].

For evaluation of construct validity, we used the FAOS as a disease-specific score and the FAAM as a region-specific score for correlation with the MAQ. Both scores had at least some evidence for score interpretation. However, none of the scores was completely valid, reliable and responsive [5, 8].

As far as we know, the MAQ is the first self-reported questionnaire that supports the assessment of ROM for ankle disorders by using images. Hence, there is no appropriate score for validation available. We decided to correlate the results of the patient-measured ROM subscale with the physician-assessed ability of dorsiflexion and plantarflexion. This unique ankle questionnaire subscale enables the MAQ to assess data which so far could only be obtained by a professional observer. With good results of construct validity for all subscales (Table 4), it can be clarified that the MAQ measures what it is supposed to measure [13, 22, 23].

For assessment of test–retest reliability, the individuals have to be tested at least twice over a period of time when the individuals’ health condition is not expected to change. After reviewing the current literature, there is no gold standard for the exact time point of determining test–retest reliability. Within a period of 2 weeks between each measure, the individual’s condition is expected to remain stable [15]. The questionnaires were received in an average of 18 days after initial consultation. The calculated ICCs indicated proof of reliability for each subscale (Table 3) [13, 15, 32]. Due to the slight delay of returned questionnaires, there is still a risk of a potential status change, which could influence the evidence for test–retest reliability. Two patients returned the questionnaires after 5 days increasing the potential risk for recall bias.

For evaluation of responsiveness, the patients were asked to complete a GPE score after 4 months regarding the change of each subscale. The results were then compared with the change in the MAQ result over the same time. Calculated SCCs indicated proof for responsiveness and the ability of the MAQ to detect individual status change [24]. To account for the relatively low SCC values (0.42–0.48), we have to point out that a majority of the patients had already undergone operative treatment 6 weeks before the first consultation and the first completion of the MAQ. It can be assumed that the effect of surgery has the biggest influence on the patients’ subjective change of ankle status. However, the improvement in the recreation phase after operations still matters and can be measured by the MAQ. Furthermore, there is no gold standard for the evaluation of responsiveness. In addition to the GPE score, a “standardized response mean” and “effect size” could be calculated for more precise determination of responsiveness [24]. Other authors suggest an objective evaluation (e.g., clinical examination) to detect potential pathology changes [13].

The correlation of the overall MAQ result with already established ankle PROMs indicated proof for score interpretation (Fig. 3).

### Limitations

It is important to note that this validation study is not without limitations.

PROMs are able to minimize the risk of interpretation bias because the information derives directly from the patients [33]. On the other hand, there is a risk of potential bias, which can be caused by non-response or incorrect completion of the questionnaires [34].

Many patients did not finish the study after a period of 4 months. This resulted in high drop-out rates for the assessment of test–retest reliability and responsiveness. In spite of that, we included more than the recommended 50 individuals [15]. Therefore, we expect only a minimal potential influence. According to Parker et al. [34], individuals can be reminded by telephone to reduce drop-out rates. However, this could cause unnecessary pressure with the risk of decreased interest to further participate in the study.

Furthermore, the patients completed the second and third questionnaire at home in another setting with potential risk for location bias. However, as shown in previous studies, we consider this aspect to be minor because the questionnaires were always self-completed [21, 23]. Another limiting factor is a potential selection bias due to missing randomization. Patients enrolled in this study might have participated only due to very good or poor postoperative results. Although having enrolled a wide range of ankle pathologies, the remaining spectrum is still large and further disabilities could be included including chronic disorders like deformities. Another important fact is that the MAQ was only validated in German and an international use requires a several study with the English version of the MAQ. Moreover, an additive validation study for cross-cultural adaption would be necessary.

## Conclusions

In conclusion, a high internal consistency, construct validity, test–retest reliability and responsiveness were achieved for the MAQ in the presented validation study. It is the first foot-and-ankle questionnaire which uses illustrations to determine ROM in a self-assessment manner.

## Additional file

**Additional file 1.** Munich ankle questionnaire.

## Abbreviations

AOFAS: Ankle Hindfoot Score
FAAM: Foot and Ankle Ability Measure
FADI: Foot and Ankle Disability Index
FAOS: Foot and Ankle Outcome Score
GPE: Global perceived effect
ICC: Intraclass correlation coefficient
LEFS: Lower Extremitiy Functional Scale
MAQ: Munich Ankle Questionnaire
PCC: Pearson correlation coefficient
PROM: Patient-related outcome measurement
RCT: Randomized controlled trial
ROM: Range of motion
SCC: Spearman correlation coefficient
TKA: Total knee arthroplasty
