# Supplementary material for: The Munich Ankle Questionnaire (MAQ): a self-assessment tool for a comprehensive evaluation of ankle disorders
Source: Eur J Med Res. 2018 Sep 28;23:46. doi: 10.1186/s40001-018-0344-7 (PMC6161467; doi:10.1186/s40001-018-0344-7)
Supplement: Supplementary file 1 — Additional file 1. Munich ankle questionnaire. [file 40001_2018_344_MOESM1_ESM.pdf]

Münchener Sprunggelenksfragebogen

(Munich ankle questionnaire)

Klinik und Poliklinik für Unfallchirurgie

Klinikum rechts der Isar

Bitte beantworten Sie jede Frage mit einem Kreuz!

Datum:	
Geschlecht:	☐ männlich ☐ weiblich
betroffenes Sprunggelenk:	☐ rechts ☐ links
Berufstätig	☐ ja, als: ____________________<br>ja, ☐ körperlich ☐ stehend ☐ sitzend ☐ gehende<br>☐ nein, falls nein: wegen Sprunggelenk: ☐ ja ☐ nein
Sport	☐ nein ☐ Joggen/Laufen ☐ kontaktloser Sport ☐ Kontaktsportart<br>☐ Hobby ☐ Amateur ☐ Profimäßig

Nehmen Sie Medikamente wegen Schmerzen des betroffenen Sprunggelenks? (4)

☐ nein (4)

☐ ja, wenn ja: ☐ bei Bedarf (2) ☐ dauernd (0)

wenn ja, welche und in welcher Dosierung:

____

____

____

Wie sehr sind Sie mit dem Behandlungsergebnis zufrieden?

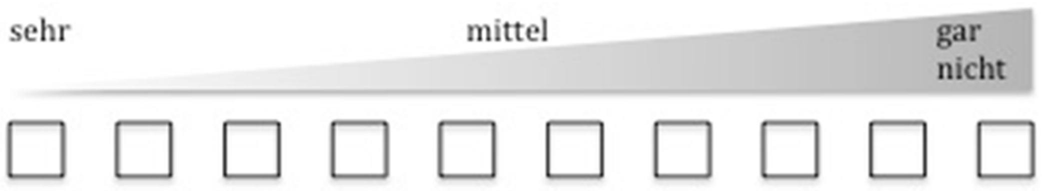

Schmerzen (30)

Wie stark sind Ihre Schmerzen...

...in Ruhe? (10)

keine – leicht – mittel – stark – unerträglich

☐ 10 ☐ ☐ ☐ ☐ ☐ ☐ ☐ ☐ ☐ 1

... im Alltag? (10)

keine – leicht – mittel – stark – unerträglich

☐ 10 ☐ ☐ ☐ ☐ ☐ ☐ ☐ ☐ ☐ 1

...bei starken Belastungen? (10)

keine – leicht – mittel – stark – unerträglich

☐ 10 ☐ ☐ ☐ ☐ ☐ ☐ ☐ ☐ ☐ 1

Alltag und Arbeit (47)

Haben Sie Schwierigkeiten beim Gehen auf...

...unebenem Untergrund? (10)

keine – wenig – mäßig – stark – unmöglich

☐ 10 ☐ ☐ ☐ ☐ ☐ ☐ ☐ ☐ ☐ 1

...ebenem Untergrund im Freien? (10)

keine – wenig – mäßig – stark – unmöglich

☐ 10 ☐ ☐ ☐ ☐ ☐ ☐ ☐ ☐ ☐ 1

...in der Wohnung? (10)

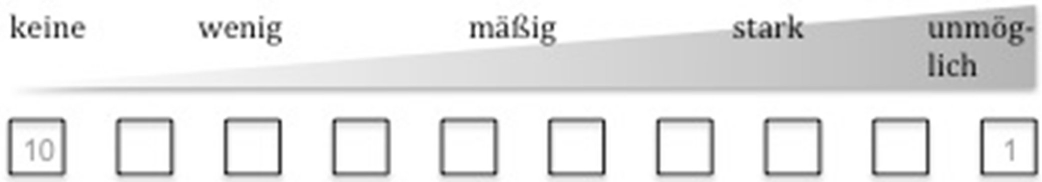

Haben Sie Schwierigkeiten beim Treppensteigen? (10)

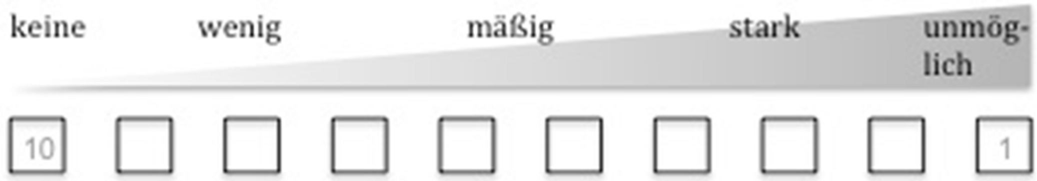

Konnten Sie Ihre alte Arbeit wieder aufnehmen? (7)

☐ alte Arbeit, uneingeschränkt (7)

☐ alte Arbeit, eingeschränkt (5)

☐ arbeitsfähig, aber nicht in alter Arbeit (3)

☐ arbeitsunfähig aufgrund Sprunggelenksverletzung (1)

Bewegung (29)

Können Sie auf den Zehenspitzen stehen/gehen? (3)

☐ nein (1) ☐ eingeschränkt (2) ☐ ja (3)

Können Sie auf den Fersen stehen/gehen? (3)

☐ nein (1) ☐ eingeschränkt (2) ☐ ja (3)

Können Sie springen? (3)

☐ nein (1) ☐ eingeschränkt (2) ☐ ja (3)

Bewegungsumfang (20)

Bitte benutzen Sie zur Ermittlung des Bewegungsumfanges Ihres betroffenen Sprunggelenkes die beiliegenden Schablonen (Abbildungen 1-2). Für das rechte Sprunggelenk benötigen Sie die Abbildung 1, für das linke Sprunggelenk die Abbildungen 2.

Wichtig: Untersuchen Sie immer den Bewegungsumfang beider Sprunggelenke, da Ihr gesunder Fuß als Bezug dient.

Abbildung 1: rechtes Sprunggelenk, ohne Belastung (aktiv)
Abbildung 2: linkes Sprunggelenk, ohne Belastung (aktiv)

Halten Sie die Schablone an die Außenseite Ihres Fuß und kreuzen Sie die Linie an, deren Position Sie mit Ihrem Fuß einnehmen können. Der Kreis stellt das Drehzentrum dar, das in etwa ihrem Außenknöchel entspricht. Achten Sie darauf, dass ihr Fuß sich nur auf und ab bewegen darf und keine seitlichen Bewegungen macht.

Ohne Belastung (Abbildungen 1 & 2):

Überkreuzen bzw. überschlagen Sie die Beine (der zu untersuchende Fuß darf keinen Bodenkontakt haben) und halten Sie die Schablone an die Außenseite Ihres Fußes. Die Ausgangsposition ist ein rechter Winkel (90°) zwischen Unterschenkel und Fuß. Ziehen Sie Ihren Fuß an bzw. strecken Sie ihn soweit es Ihnen möglich ist. Kreuzen Sie die Stellen an, die Ihren Maxima entsprechen. **Wichtig**: Der Winkel der Fußsohle zum Unterschenkel ist entscheidend, nicht der Winkel der Zehen.

Abbildung 1

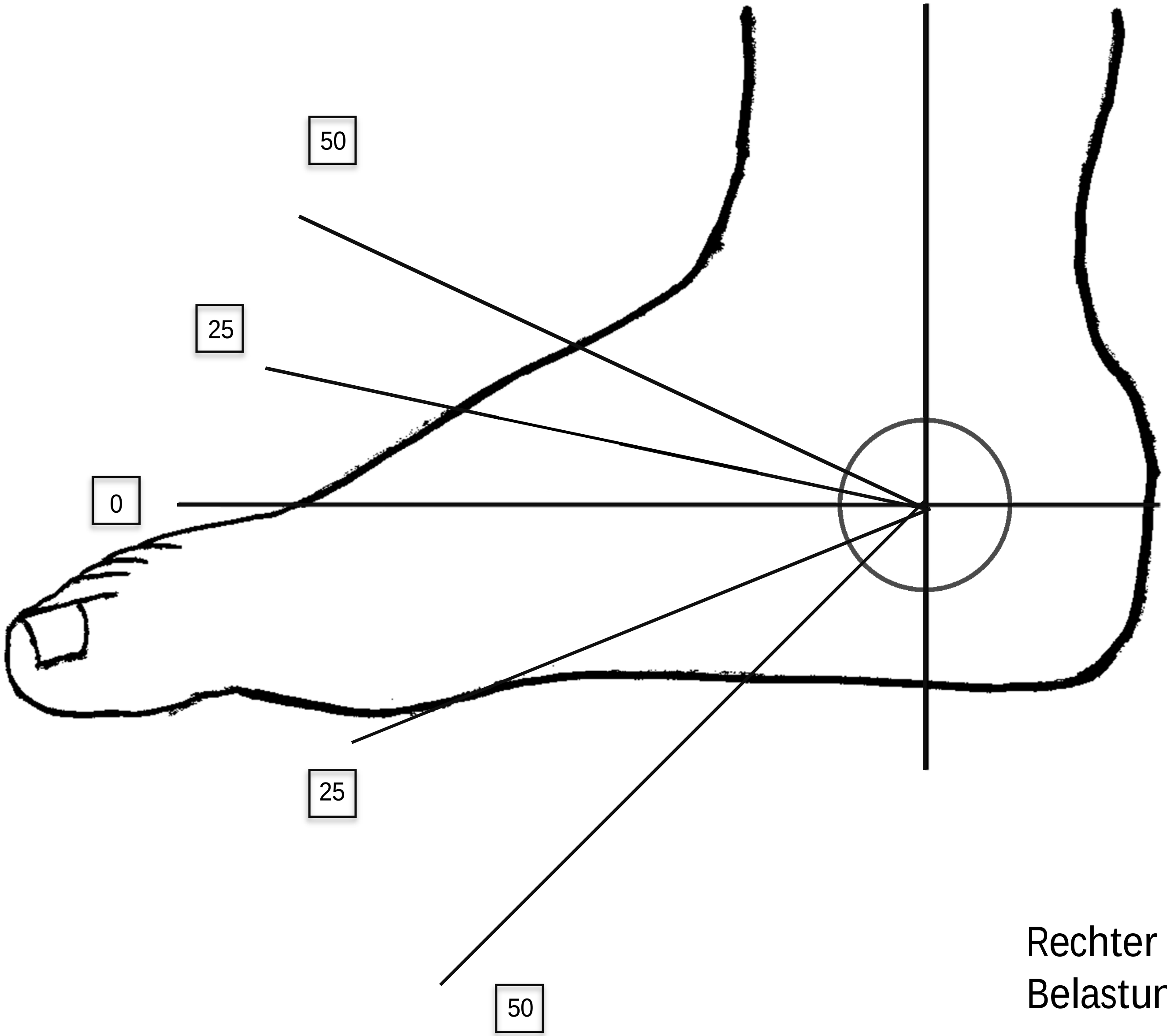

Rechter Fuß, ohne
Belastung (aktiv)

Abbildung 2

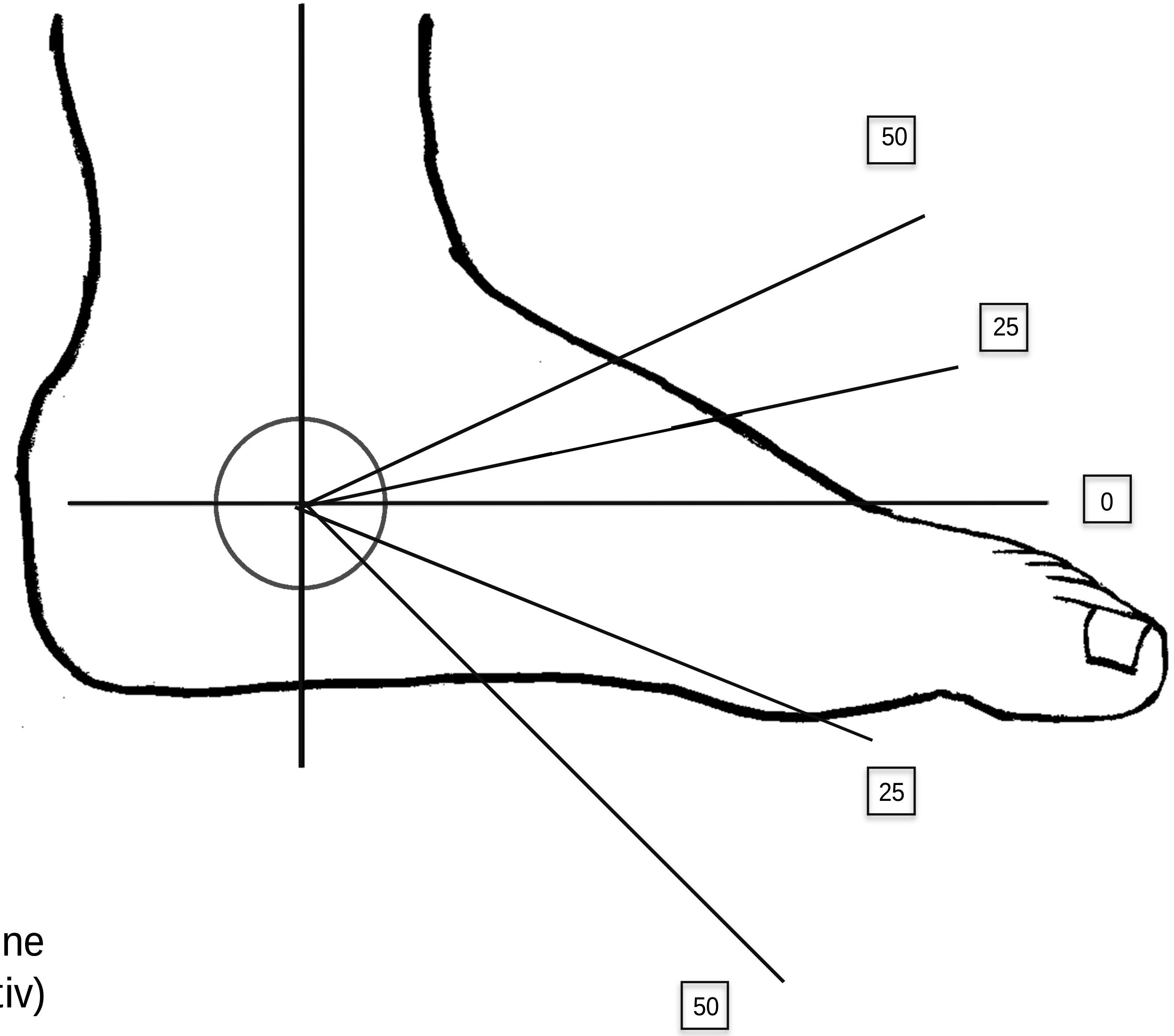

Linker Fuß, ohne
Belastung (aktiv)
